# Applying Collaborative Care Model on Intensive Caregiver Burden and Resilient Family Caregivers of Patients with Mental Disorders: A Randomized Controlled Trial

**Published:** 2020-01

**Authors:** Mohammad Zoladl, Solaiman Afroughi, Khairollah Nooryan, Shirali Kharamin, Amin Haghgoo, Yaghoub Parandvar

**Affiliations:** 1 School of Nursing and Midwifery, Yasuj University of Medical Sciences, Yasouj, Iran.; 2 Department of Biostatistics and Epidemiology, School of Health and Social Determinants of Health Research Center, Yasuj University of Medical Sciences, Yasuj, Iran.; 3 Department of Psychology, School of Medicine, Yasouj University of Medical Sciences, Yasuj, Iran.

**Keywords:** *Caregiver Burden*, *Family Caregivers*, *Mental Disorders*, *Participatory Care Model*, *Resilience*

## Abstract

**Objective:** Psychological education for families in the form of a model is one of the effective approaches in managing problems caused by mental health problems. The present study aimed to determine the effect of using the participatory care model on the caregiver burden and resilience of home caregivers of patients with mental disorders.

**Method** **:** In this clinical trial, 66 households with psychiatric patients hospitalized at Shahid Rajaee Psychiatric Hospital in Yasuj during 2014-2015 were selected and assigned into 2 groups of experimental and intervention based on convenience and simple random sampling. The data of this study were gathered by Novak & Guest (1989) Caregiver burden and Sixbey (2005) Resilience Questionnaire before and after intervention. Participatory care model was performed for 12 ninety-minute sessions in the intervention group. No intervention was provided to the control group during the study period. SPSS software (version 21) was used to run the descriptive and inferential statistics.

**Results: **Chi-squared test showed that the caregiver burden was significantly lower in the experimental group than in the control group after the intervention (P = 0.0001). Following the intervention, increased resilience and all its components were observed in the experimental group compared to the control group. According to the independent t test and Mann-Whitney U, the 2 groups were considerably different (P < 0.05).

**Conclusion: **The application of the participatory care model efficiently increased resilience and decreased the intensity of the caregiver burden on the home caregivers of patients suffering from mental disorders.

The family is a hidden health care system in the health spectrum where the psychiatric patient lives in and is being cared for (1). Low life expectancy and quality of life are always seen in the family caregivers of mental patients (2). Providing care for patients in the family has led to great challenges for families (3). Families are not completely aware of the available resources to handle problematic and complex behaviors of patients, but they typically are wrestling with feelings of guilt, confusion, grief, and emotional problems (4). Caregiver burden is preponderantly used as an indicator of the caregiving experience; however, much disagreement remains on what the term entails and how it should be utilized. According to previous studies, sources of psychiatric stress and caregiving in the family of psychiatric patients are as follow: Tolerance or lack of autonomy; increased susceptibility and acceptance of intolerable medical regimens in the patient; the struggle of living with these patients because of unexpected and surprising changes in the lives of family members; providing constant care for the mentally ill at home; constant hospitalization because of the relapse of the disease; not having a constant contact with a doctor; arbitrary discontinuation of medication; social isolation; lack of social support; loss of working capability; decreased family economic capacity; lack of care and necessary training to face destructive manners of a patient; facing the legal issues of the patient's behavior in the community; the shame of knowing the presence of the disease in the family and making changes in the interpersonal relationships in the family (6).

Resilience is one of the strategies of check index that these individuals can use to promote mental health and to cope with stress and reduce the pressure it poses (7). Resilience means the ability to cope with difficult situations and have more flexible responses to the pressures of life, so that one can improve their social performance, which leads to positive reaction in difficult and stressful situations (2). There is a significant inverse correlation between mental disorders and resilience (Khakpour & Mehrafarid, 2013). Resilience training programs, especially in families, improve relationships with others, increase positive emotions, self-esteem, self-management, and reduce negative emotions, stress and depression (8). Psychiatric nurses should, in addition to providing care services, be able to train the patient and his family for a new life and adapt to potential changes (9). One of the best ways to achieve this is to use theory and model. Work based on a nursing model helps to better assess the status of patients, provide meaningful and useful communication between patients and nurses, determine care objectives, improve the quality of care, and guide and clarify activities (10). Patients in developed countries are educated by social workers to learn to self-manage and reduce the consequences of illness, which leads to their informed participation in their care program. For the first time in Iran, in 2001, a collaborative care model was designed by Isa Mohammadi in Babol to control blood pressure. Participatory care is the systematic and rational process of effective and balanced communication, which, based on this model, in the care process, the quality and the type of relationship between the 2 sides of the relationship is of crucial importance (11). Studies on the quality of life of hemodialysis patients have confirmed the effectiveness of this model (12) on children with major thalassemia (13) and chemically injured patients with chronic pulmonary disease (14). Identifying and prioritizing the care needs of the families with psychiatric patients, planning properly, and applying nursing models in providing and meeting the needs of the families with patients were the tasks of nurses. Several studies reported lack of support from family caregivers and lack of attention to their needs (15, 16). Evidence suggests that nurses are accustomed to seeing the world through a professional perspective, which limits their thinking and ultimately their performance. For the desirable performance, the world needs to be seen through the patients' eyes (17). This is crucial in nursing education, because nursing education empowers nurses in responding to clients’ needs and providing client-centered care in clients' perspective. Therefore, since the participatory care model is used to deliver mental health services that can be accompanied with the help of the mental health team and given the importance of the subject, the present study tried to show how the application of participatory care model affects the resilience of the family of psychiatric patients during 2015-2014.

## Materials and Methods

***Participants***

This clinical trial was conducted on family caregivers of patients suffering from mental disorders who had a 3-month history of hospitalization in Shahid Raja’ee Psychiatry hospital in 2014-2015 in Yasuj. The sample size was determined based on the previous studies, with 66 households (246 people) (18, 19). 

***Study Participants and Sampling***

With the permission of Vice Chancellor for Research and Treatment Affairs of the University, the contact information of the patients was extracted from their medical records, then the family caretakers were invited to participate in the study and informed consent was obtained. The samples randomly and through convenience sampling were assigned into 2 groups of experimental (N = 33) and control (N = 33), and a list of all eligible home caregivers was compiled and numbers were assigned to them. Then, a random number was selected from the random number table and the 2 right digits matching the number of people in the list were considered up to completion of the sample size in the experimental group and the remaining people were selected as the control group. In this study, the mean total score of each household (the first-degree relative of the patient who was the main caregiver and lived in the same house with the patient) was calculated as a sample in data collection instruments.

***Inclusion Criteria***

The study inclusion criteria were as follow: age range of 15-75 years, being able to read and write, being the main caregiver, not suffering from a mental illness, and providing written informed consent to participate in the study.

***Exclusion Criteria***

The study exclusion criteria were as follow: the choice of leaving the study at any time, unfinished questionnaires, relocation, and absence in the training sessions. 

***Instruments***

1. Data collection instrument included a checklist of demographic information on age, gender, education, marital status, caregiver's relationship to the patient, occupation, the type of mental disorder, patient care period, and family-based educational needs assessment checklist, including 29 questions in the field of knowledge (7 items), attitude (7 items), and performance (15 items). The checklist’s questions were extracted from similar studies, questionnaires, and available books. Five faculty members verified the content and face validity of the checklist. The reliability of the initial needs assessment checklist was obtained with the Cronbach alpha of 0.76.
2. The 24-Item Caregiver Burden Inventory (CBI) was developed by Novak and Guest in 1989 to measure the objective and subjective caregiver burden and measure mental caregiver burden with more precision. In a 5-point scale, the person determines the degree to which he/she experiences the mentioned situations. The 5 subscales of this questionnaire include time-dependent caregiver burden (Questions 1 - 5), which are related to the time constraints of the caregiver. Evolutionary Caregiver Burden (Questions 6 - 10) investigates whether the caregiver feels that he or she develops less than his peers. Physical Caregiver Burden (Questions 11-14) describes the caregiver's feelings about physical threats or injuries. In addition, the social caregiver burden (questions 15 to 19) generally investigates the feelings of role conflict of the caregiver, disputes with other family members about the client, and in general, the feeling of lack of appreciation and rejection. Furthermore, emotional caregiver burden (Questions 20 - 24) measures the negative emotions of caregivers to the patients. In response to each question, the participants selected one of the choices of totally false, false, somehow correct, correct, and completely correct, with the scores of 1 to 5, respectively. Accordingly, the scores ranged from 24 to 120, with scores of 24 to 47 showing slight caregiver burden, score 48 to 71 moderate caregiver burden, 72 to 95 intense caregiver burden, and 96 to 120 very intense caregiver burden (20). In the study of Waller et al (2015), the questionnaire's Cronbach's alpha was calculated as 93% (21). According to the study by Abbasi et al (2013), the content validity index of the questionnaire was investigated for relevance (91.8%), clarity (90.2%), and simple and verbal expressions (93.6%) by 10 faculty members. In total, the CVI of this questionnaire was 91.86%. Also, reliability of the questionnaire was reported with Cronbach's alpha coefficient of 90% (22).
3. Family Resilience Inventory: This 66-item scale was created by Sixbey in 2005 to measure family resilience based on the Walsh family resilience model. This questionnaire measures the family resilience in 6 areas of family resilience and problem solving (28 questions), use of socioeconomic resources (8 questions), maintaining a positive perspective (6 questions), family relationship (6 questions), family spirituality (4 questions), and the ability to create meaning for difficulty and hardships (4 questions). This questionnaire is scored from 1 to 4, with 1 as strongly disagree to 4 as strongly agree. The minimum score obtained on this scale is 66 and the maximum 204, with the high score on this scale reflecting the high resilience of the family and the low score representing low family resilience (22).The psychometrics of this scale was confirmed by Sixbey (2005). Buchanan (2008) reported the total reliability of the Family Resilience Scale with to be 0.96 (23). This questionnaire was standardized by Sadat Hosseini et al (2013) in Iran. The total reliability of this scale was reported to be 0.93 (24).

***Intervention Program***

The main interventions in the intervention group were made by motivation, preparation, engagement, and evaluation. In this model, after needs assessment, the social workers were informed about important issues that they needed to cover with the patients and on which the statistical analysis was done, and all members of the team, including clinical psychologists and psychiatric nurses, participated in the study enthusiastically. The primary reading checklist was used to measure the educational needs. Afterwards, the patients’ problems were defined by a psychiatric nurse. Then, preparation and engagement were performed according to the participatory care model during 12 weeks in the hospital. According to the patients’ needs and the type of problems, 8 training sessions were established based on the arrangement of the primary need assessment checklist and 4 collaborative visit follow-ups. First, the patients were familiarized with care problems, current conditions, the risks, and complications of stress, and they were encouraged for more engagement in self-care. Furthermore, at this stage, the caregivers were briefed about the aimes of the sessions, duration and place of the sessions, teaching method, and participants. The client was engaged in the second phase of the model which started with collaborative educational visits in 8 sessions covering the following issues:

***A) Collaborative Educational Visits (8 sessions) (*** Table1 ***)***

All collaborative educational visits were introduced and made through speech, brochures, and questions-answers session within 60 to 90 minutes. 

***B) Collaborative Visits: First to Fourth Follow-ups***

Each visit session lasted 30 minutes, with one-week break in between. In these visits, the problems of the patients were checked and the positive and negative consequences of the educational actions were examined. The [articipants were given instructions on how to solve problems. After the assessment phase, a participatory care model was adopted one month after the intervention in the intervention group and in the controls. After the assessment, the control group was briefed about the positive progress in the intervention group and all educational content and follow-up visits by a leaflet.

***Ethical Considerations***

The Code of Ethics 92.12.3.12 of the present study was issued by the Vice-Chancellor of Yasouj University of Medical Sciences. This study was approved by the Iranian Registry of Clinical Trials IRCT2015060622580N1 of Iran University of Medical Sciences. At the beginning of this study, the research nurses explained the study purpose, methods, and procedures to all participants and then started conducting the data collection and intervention program after having obtained written informed consent. During the study period, all participants were given the permission to leave the study at any time. To ensure participant privacy, all data were encoded and used only for research purposes.

***Data Analysis***

Data were analyzed using SPSS version 21. Demographic variables were analyzed using descriptive statistics (frequency, mean). Kolmogorov-Smirnov test was used for the accuracy of the normal distribution. Also, independent t test was used to compare the means of variables. Chi-squared test was applied to compare the frequency distribution of qualitative variables between the experimental and the control groups. The marginal homogeneity test was employed to measure the variability of the multicategorical variables before and after the intervention. Moreover, the Mann-Whitney U test was used to compare the means of the skewed variables between the 2 groups.

## Results

***Participant Characteristics***

Based on Table 2, chi-squared test did not indicate any significant differences between the 2 groups in demographic information including sex, relationship to the patient, education, occupation, and the type of mental disorder (p > 0.05). Most participants of both groups were females. The most frequent level of education was high school diploma in the experimental group (n = 34; 26.77%) and Bachelor's degree in the control group (n = 52; 45.62%). The lowest level of education was middle school education in both groups, and most participants in both groups were employees. Most caregivers of mental patients were patients' spouses in both groups and the fewest caregivers were sisters in both groups. Moreover, the most common psychiatric disorders in the family of patients were anxiety disorder (n = 10; 15.2%) in the experimental group and mood disorder (n =11; 16.6%) in the control group, and the least mental disorder in both groups was psychosomatic disorders. 

According to Table 3, the mean age of caregivers was (33.95±13.98) in the experimental group and (3.21±34.85) in the control group. Also, the mean duration of patient care in the experimental group was 12.51 months and the mean duration of patient care in the control group was 12.02 months. Mann-Whitney U test did not indicate a significant statistical difference between the mean of the 2 groups in age and duration of patient care (p > 0.05). Therefore, it can be concluded that the research units of the experimental and the control groups were similar in the underlying variables of age and duration of patient care.

***Pretest and Posttest Results***

The inferential findings of the present study indicated that at the beginning of the study the intensity of caregiver burden belonged to the middle and intense categories in both groups, and the chi-squared test results did not confirm a significant difference between the intensity of the caregiver burden in both the control and experimental groups (P < 0.05) (Table 4).

After intervention, none of the participants in the intervention group had severe caregiver burden and most of them were in the mild category (48.5%), while the control group was classified as having mild (22.7%), moderate (21.2%), and severe (6.1%) caregiver burden, respectively. Furthermore, chi-squared test results confirmed that after intervention, the severity of caregiver burden in the intervention group was significantly lower than that of the control group (P = 0.0001) (Table 5). Although the participants were randomly assigned to the experimental and control groups prior to the intervention, the resilience and all its components in the intervention group were greater than the control group at the beginning of the study. According to the results of independent t test, the resilience mean and its 2 components (family relationship and problem-solving and maintaining a positive outlook in the intervention and control group) were significantly different at the beginning of the study (p < 0.05). However, the mean of the other resilience components of the 2 groups were not significantly different at the beginning of the study (P < 0.05). After intervention, the resilience and all its components in the intervention group increased compared to the control group. Also, independent t test and Mann-Whitney test U results showed a significant statistical difference between the 2 groups in the resilience dependent variable and all its components (P < 0.05). 

## Discussion

The present study was conducted to determine the effect of using participatory care model on the intensity of care and resilience of home caregivers of patients with mental disorders in Yasuj. The descriptive findings of this study showed no significant difference in the demographic information between the experimental and the control groups, which was similar to the results of the study by Qomi et al (18).

The results of this study indicated that the intensity of caregiver burden was high in both groups at the beginning of the study. After intervention, the intensity of caregiver burden decreased in both groups, but this decrease was significantly more in the experimental group. Caregivers of patients are forced to meet their needs as well as the needs of the patients simultaneously, which leads to excessive burden on physical, emotional, social, and economic aspects of their life (33). Chan (2004) reported that when caregivers have a clear understanding of their patients’ illness, symptoms, and condition, their stress and anxiety are reduced and their ability to cope with their problems increases (34). Grawe et al (2006) argued that the family psychological education decreased the care burden of caregivers of schizophrenic patients efficiently (35). According to Bernard et al (2006), the feeling of pressure or family burden was decreased considerably by family psychological education in bipolar patients after intervention and a year afterwards (23).The results of these studies were consistent with those of the present study. 

The effective results of the participatory caregiver model were consistent with the study of Emami et al (2015) who reported the efficiency of family psychosocial training on the psychological health of home caregivers (27). In this study, home caregivers were trained with coping strategies for adaptation, communication, problem-solving, mental illness management, and medical and nonmedical treatments with psychiatric patients in the form of participatory care model, reducing the intensity of caregiver burden in these patients. Cheraghi et al (2011) investigated the needs of psychiatric patients and their families and indicated that the family of these patients need postdischarge training and personal and family counseling on drug use, social, vocational, and occupational skills training as well as training to raise awareness of the society and reduce social stigma and social discrimination (36). The patients opting for drug therapy and psychotherapy methods have the double benefit of recovery and reducing caregiving burden.

The results of this study showed that due to the random assignment of the groups, the resilience scores of the experimental group were higher than the control group at the beginning of the study, and the findings of the study by Carlton et al (2006) confirmed the result of the present study (31). The results of this study showed that the resilience of the samples in the experimental group after intervention was significantly different from the preintervention stage. Findings of the study conducted by White et al (2002) indicated that training-based interventions reduce stress and increase the resilience of home caregivers in dialysis patients (37). The resilience scores of the home caregivers of the experimental group increased after the implementation of the participatory care model. In the studies conducted by Freiburg et al (2007), Prince-Ambury (2008), and Qomi et al (2013), the increase in resilience in the experimental group was proved by training-based interventions (18, 38, 39). The results of Hatice's study indicated that supportive training improves resilience and its components in home caregivers of patients with stroke (40). Reviewing the literature showed that increasing protective factors and decreasing risk factors are effective in boosting resilience (41), which is consistent with the results of the present study. Other reasons for increased resilience scores can be the use of problem-solving methods and decision-making, life skills training, communication with others, and the explanation of the resilience structure in resilience training sessions. With regards to the positive impact of implementing a participatory care model, the following studies can be considered.

Sullivan et al (2007) reported that anxiety disorder was enhanced considerably following the implementation of participatory care model (42). According to Hegel et al, participatory care affected management depression in the elderly compared to the routine care (43). Graham argued that in contrast to routine care, participatory care led to improved depression in the elderly (44). Azadi et al (2006) explored the pattern of participatory care to enhance the quality of life of patients suffering from coronary artery disease and reported that this model efficiently enhances the quality of life of those individuals (45). Also, Nayeri et al confirmed the positive influence of participatory care model on the quality of sleep in patients suffering from heart failure. Thus, some of the implications of these researches on the use of participatory care model were confirmed by earlier studies (46). In a meta-analysis, Gabriel et al (2014) reported that the collaborative care model is effective in improving patients with complicated medical-psychiatric conditions (47). There have been many reviews of collaborative care for the management of patients with chronic illnesses. A randomized controlled trial in 14 primary care clinics in an integrated health care system in Washington State studied patients with depression and poorly controlled diabetes, coronary heart disease or both and concluded that compared with the usual care, collaborative care involving nurses led to significant improvement in the management of depression and chronic diseases. (48) In addition to clinical effectiveness, collaborative care has also been demonstrated to be cost-effective (49). England has been making efforts to scale up integrated/collaborative care. A recent report for the Department of Health in England covering 16 integrated care pilots (ICPs), some of which specifically included some mental health and dementia services, concluded that where there had been perceived benefits, facilitators to ICP success included strong leadership and pre-existing relationships at a personal level across organizations, shared values, collective communicated vision, investment of effort in widespread staff engagement and the provision of education and training specific to service change (50). Collaborative care is an effective model for integrating behavioral (mental) health care into primary care medical settings. Also, it aims to improve the physical and mental health of those with mental illness, and specifically aims to develop closer working relationships between primary care and specialist health care.

## Limitation

The lack of comparison of this model with other educational methods for home caregivers of psychiatric patients due to lack of time was among the limitations of this study. Therefore, it is suggested that researchers make such comparison and investigate the effect of this model on improving the quality of life of home caregivers of psychiatric patients.

**Table 1:** Content of Intervention Group Training Sessions Using Participatory Care Model (25-30)

**Session / Topic**	**The content of the session**
First session: ow the family members are involved in the patients' counseling	Familiarity with training sessions, familiarity with principles and criteria of mental health, explaining and clarifying the role of the family in developing and maintaining the health of family members, explaining and clarifying the role of the family in the rehabilitation of mental patients, familiarity with the causes and factors of mental disorders, the scientific explanation of common misconceptions and attitudes about mental illness
Second session: awareness of mental illnesses	Familiarity with definition of mental illnesses, etiology, symptoms and signs, types, procedures and prognosis of the disease, identifying and managing the warning signs, tolerance to permanent signs of the disease.
Third session: awareness of medication methods for patients with mental disorders	Familiarity with the benefits of drugs, drug complications, appropriate use of medications, reducing drug side-effects
Fourth session: nonmedication therapies for patients with mental disorders	Familiarity with nondrug treatments, exercising, relaxing and other anxiety- reducing methods, and how to fill the patient's leisure time
Fifth session: psychological communication	How to communicate with the mental patient, how to provide care and treat the signs and symptoms of illness, illusion, delirium, aggression and aggressive states in patients, immunizing the patient's living environment, preventing aggressive and suicidal states, and how to refer to the related medical centers and other support centers for psychiatric patients and their families
Sixth session: how to manage the stress and excitement	Investigating burden factors, Ways to cope with stress, Ways to cope with anxiety
Seventh session: how to solve problems and make decisions	Problem-solving process, Problem-solving steps , Self-awareness, The logical decision-making steps, obstacles of problem solving
Eighth session: how one becomes resilient	The nature of resilience, Factors contributing to resilience, how one becomes resilient, Recommendations for resilience

**Table 2:** Comparison of Demographic Variables in Experimental and Intervention Groups in the 2 Groups

**Specification** **of caregivers**		**Cases**		**Controls**			**X2**		**P-value**
		**Percentage**	**Frequency**		**Percentage**	**Frequency**			
Sex	Male	45.66	58		47.36	54	0.070	0.792	
	Female	54.33	69		52.67	60			
	Total	100	127		100	114			
Relationship to mental illness patient	Father	21.27	27		24.56	28	1.029	0.960	
	Mother	22.84	29		18.42	21			
	Sister	3.91	5		4.40	5			
	Brother	6.28	8		4.40	5			
	Child	4.75	6		4.40	5			
	Spouse	40.95	52		43.82	50			
education	Elementary	5.51	7		6.14	7	17.555	0.007	
	Middle- school	4.72	6		1.75	2			
	High school	18.11	23		8.77	10			
	Diploma	26.77	34		16.67	19			
	Associate's Degree	16.53	21		15.79	18			
	Bachelor's degree	22.85	29		45.62	52			
	Master's degree and higher	5.51	7		5.26	6			
Employment status	Unemploye d	4.69	6		7.01	8	6.075		0.299
	Self- employed	22.05	28		21.05	24			
	Employee	23.65	30		28.07	32			
	Housekeep er	22.05	28		21.92	25			
	Pupil	11.81	15		10.52	12			
	Student	15.75	20		11.43	13			
Disorder of the mental patient	Mood disorders	13.6	9		16.6	11	1.344		0.719
	Anxiety Disorders	15.2	10		9.1	6			
	Psychosom atic disorders	7.6	5		9.1	6			
	Schizophre nic disorders	13.6	9		15.2	10			

**Table 3:** Frequency Distribution of Intensive Caregiver Burden in Home Caregivers of Mental Patients before and after Study in Experimental and Control Group

	**Before intervention**		**After intervention**	
**Group** **Intensive ** **caregiver** **burden**	**Intervention ** **group**	**Control group**	**Intervention ** **group**	**Control group**
**Mild**	0(%0)	0 (%0)	32 (%48.5)	15 (%22.7)
**Medium**	20(%13.3)	14 (%21.2)	1 (%1.5)	14 (%21.2)
**Intense**	13(%19.7)	19 (%28.8)	0 (%0)	4 (%6.1)
**Very intense**	0(%0)	0 (%0)	0 (%0)	0 (%0)
**Total**	33(%50)	33 (%50)	33 (%50)	33 (%50)
**2** **χ**	2.18		21.42	
**P-value**	0.14		0.0001	

**Table 4:** Mean and Standard Deviation of Resilience Scores and its Components in the Home Caregivers of Mental Patients at the Beginning of the Study in the 2 Groups

**Group**		**Intervention group**	**Control group**	**T**	**P-value**
**The statistics**		**M±SD**	**M±SD**		
**Variable**					
Resilient		21.62±7.34	311.68 ±4.33	-4.32	0.0001
Resilience components	Family relationship and problem solving	16.50±4.21	65.86±3.3	-4.69	0.0001
	Benefit from economic/ social resources	71.55±1.61	71.21±1.22	-1.22	0.228
	Maintain a positive outlook	31.24±1.34	21.93±1.04	-3.49	0.001
	Family link	31.1±1.34	21.58±1.02	-0.84	0.41
	Family spirituality	8.7±1.1	8.14±0.95	-1.16	0.249
	The ability to create meaning for difficulty	6.44±0.85	6.4±1.01	-0.23	0.824

**Table 5:** Mean, Standard Deviation, and Mean Rank of Resilience Scores and Its Components in Home Caregivers of Psychiatric Patients after Intervention in the 2 Groups

**Group**		**Intervention group**		**Control group**		*Z **Mann-Whitney U** **T	**P-value**
**The statistics**		**M±SD**	**Mean ** **rank**	**M±SD**	**Mean ** **rank**		
**Variable**							
Resilience		161.91±5.75	50	311.24±6.57	17	-6.98*	0.0001
Resilience components	Family relationship and problem-solving	18.30±3.06	50	65.48±4.04	17	-6.98*	0.0001
	Utilizing social service resources	32.76±1.38	50	61.11±1.15	17	-6.98*	0.0001
	Maintain a positive outlook	71.68±1.08		21.97±1.17		-18.27**	0.0001
	Family link	71.73±1.24	49.97	21.73±0.74	17.03	-6.98*	0.0001
	Family spirituality	21.52±0.98		08.24±1.04		-15.38**	0.0001
	The ability to create meaning for difficulty	9.20±0.75		6.98±1.42		-7.6**	0.0001

## Conclusion

The present study indicated the effectiveness of participatory care model as an efficient and low cost method for decreasing the caregiver burden intensity and increasing the level of resilience of home caregivers of psychiatric patients. Therefore, reducing the caregiver burden and increasing the resilience of home caregivers 

are important in providing quality care services to psychiatric patients and better managing the care process, and increasing the ability of families to deal with upcoming problems. Therefore, due to the ease of acceptance of participatory care model by family members, this model can be used to promote mental health and help cope with stress and have more flexible response to life pressures as well as enhance social functioning of individuals.
